# Co-administration of rimonabant prevents glucose intolerance in Sprague-Dawley rats treated chronically with lopinavir/ritonavir and zidovudine: an experimental study design

**DOI:** 10.11604/pamj.2023.45.6.21541

**Published:** 2023-05-03

**Authors:** Brian Lishenga Makamu, Peter Waweru Mwangi, Frederick Okonji Bukachi

**Affiliations:** 1 Department of Medical Physiology, Egerton University, P.O Box 536, 20115 Egerton, Kenya,; 2 Department of Medical Physiology, University of Nairobi, P.O Box 30197, 00100 Nairobi, Kenya

**Keywords:** Antiretroviral therapy, rimonabant, metabolic syndrome, hyperglycemia, endogenous cannabinoids, HIV, protease inhibitors, nucleoside reverse transcriptase inhibitors

## Abstract

**Introduction:**

treatment of HIV infection with Protease Inhibitors (PIs) and Nucleoside Reverse Transcriptase Inhibitors (NRTIs) can lead to insulin resistance and changes in body fat distribution. Overactivity of the endogenous cannabinoid system produces similar disturbances in metabolic syndrome within the general population. However, Cannabinoid receptor type 1 antagonism, in both human and animal studies, reverses many of these biochemical and physical derangements observed in the metabolic syndrome.

**Methods:**

using an experimental study design, fifteen adult male Sprague-Dawley rats housed under standard conditions were randomized into three groups; Control, combined Anti-Retroviral Therapy (cART) only and cART + rimonabant. Drugs were administered daily by oral gavage for four weeks. After four weeks, insulin tolerance tests were conducted, the rats were euthanised and fat depots were excised and weighed. Experimental data were analysed using STATA 16.0 with the significance level set at p<0.05. The Shapiro-Wilk test determined normalcy. In cases of significance, post hoc analysis was performed by either the Dunn test or the Tukey HSD test.

**Results:**

Sprague Dawley rats treated with cART + rimonabant demonstrated better insulin sensitivity (p = 0.0239) and lower body weight (p = 0.044) than rats treated with cART alone. They had leaner body composition with 58% less adiposity than cART-only rats.

**Conclusion:**

the study results suggest a role for the endogenous cannabinoid system in cART induced metabolic derangements and physical changes. Future studies can directly assay ECS activity in cART associated metabolic syndrome.

## Introduction

Treatment of HIV infection with combined antiretroviral therapy (cART) regimens containing the Protease Inhibitor (PI) combination of Lopinavir/Ritonavir (LPV/r) as well as the Nucleoside Reverse Transcriptase Inhibitors (NRTIs) Zidovudine (AZT) can lead to insulin resistance along with changes in body fat distribution [1,2]. The resulting derangements, such as hyperglycaemia, hyperinsulinemia, elevated levels of soluble Tumour Necrosis Factor α, high triglyceride levels, low high-density lipoprotein cholesterol levels and low adiponectin levels, are similar to those observed in the Metabolic Syndrome among the general population [3,4]. Overactivity of the endogenous cannabinoid system (ECS) among the general population occurs in hyperglycaemic and obese states [5]. In both human and animal studies, blockage of the ECS by Cannabinoid Type 1 Receptor (CB1R) antagonists such as rimonabant reverses many of the biochemical derangements and physical changes observed in the Metabolic Syndrome [6]. CB1R antagonism results in a reduction in body weight, serum insulin and glucose levels [7,8].

There is a high degree of convergence and similarity in the mechanisms by which antiretroviral drugs and ECS overactivity produce physical changes and metabolic derangements. Protease Inhibitors decrease pancreatic insulin secretion, bind to and block GLUT 4 as well as alter its expression in adipose tissue and reduce expression of the adipokines adiponectin and leptin [9-11]. Adiponectin resistance or a reduction in serum adiponectin levels is associated with insulin resistance. NRTIs, on the other hand, diminish whole-body glucose disposal [12]. ECS upregulation activates similar mechanisms in both animal and human studies [13,14].

In this study, we hypothesised that blocking of endogenous cannabinoid activity in the context of antiretroviral therapy would ameliorate cART induced hyperglycaemia and insulin resistance. Our study objectives were: to determine the effect of rimonabant on insulin tolerance in LPV/r and AZT treated Sprague-Dawley rats and to determine the effect of rimonabant on fat distribution in LPV/r and AZT treated Sprague-Dawley rats. At the end of 4 weeks, Sprague Dawley rats treated with a combination of LPV/r + AZT + rimonabant showed increased insulin sensitivity as well as less body weight gain compared to Sprague Dawley rats treated with LPV/r + AZT only.

## Methods

**Chemicals used:** lopinavir and ritonavir (4: 1 ratio, respectively) fixed-dose combination tablets manufactured by Mylan laboratories Ltd. (Maharashtra, India). Zidovudine tablets were from Hetero drugs Ltd., (Hyderabad, India). The District Aids and STI Coordinator (DASCO), Nakuru, Kenya, donated both antiretroviral drugs. Rimonabant hydrochloride (rimonabant, 99% purity) purchased from Clearsynth Labs Limited., (Mumbai, India).

**Preparation of drugs and dose calculation:** a single tablet of Lopinavir/ritonavir, (200mg/50mg) was completely dissolved in 10ml of drinking water to yield a concentration of 20/5mg/ml. For Zidovudine, a single tablet (300mg) was dissolved entirely in 30mls of drinking water to yield a solution concentration of 10mg/ml. Dosages to be administered for the antiretrovirals were calculated based on the dosing guidelines for ART drugs in adult HIV patients. That is, for Lopinavir/ritonavir 800 mg/200mg per day or 13.33/3.33mg/kg/day and for Zidovudine, 600mg per day, or 10mg/kg/day assuming a bodyweight of 60kg. Using body surface area normalisation, this translated to a dosage of approximately 80mg/20mg/kg/day of LPV/r and 60mg/kg/day of AZT in rats [15]. Rimonabant hydrochloride tablets were dissolved in dimethyl sulphoxide (DMSO) by gentle shaking followed by dilution with Tween 20 and saline (2% DMSO, 1% Tween 20, 97% saline) to a final concentration of 1mg/ml. It then was administered at a dose of 3mg/kg/day arrived at based on dose-response curves developed previously [16]. Rats were weighed every day, followed by the administration of individualised doses of the reconstituted drugs between 0900h and 1100h by oral gavage for four weeks.

**Experimental animals:** fifteen Sprague Dawley rats (*Rattus norvegicus*) weighing between 300 and 350 g at the beginning of the study were purchased from the Department of Biochemistry, University of Nairobi and housed in communal cages at the Department of Medical Physiology, University of Nairobi. The rats were randomized into three groups; Control, combined Anti-Retroviral Therapy (cART) only and cART + Rimonabant. The randomization scheme was generated by the Randomization website. using the method of randomly permuted blocks. The rats were also randomly selected for drug administration and experimental procedures. Sprague Dawley rats were selected because of their suitability for hormone profile studies and ease of handling. The rats were fed with standard rat chow (Unga Feeds, Nairobi, Kenya) and provided with water ad libitum. Rats were habituated to handling and testing procedures under a 12:12-h light-dark cycle (lights off at 1800h) for two (2) weeks before testing. Dry wood shavings were used as beddings and changed daily.

The Postgraduate Research Committee, Department of Medical Physiology, School of Medicine, University of Nairobi, approved the study protocol. Rats were handled by following the U.S National Research Council Guide for the Care and Use of Laboratory Animals.

### Experimental protocols

**Insulin tolerance test:** Sprague Dawley rats were randomised into three groups of 5 rats each. Group 1 served as the control and received a weight-matched volume of 0.5% pharmaceutical grade starch solution. Group 2 rats were treated with LPV/r + AZT while group 3 received LPV/r + AZT + Rimonabant. The insulin tolerance test (ITT) was conducted, as described by Beguinot [17]. Briefly, following a five-hour fast (at time 0), the tip of the tail was nicked with a scalpel blade. A drop of blood was expressed onto a glucose test strip and measured with a glucose meter (Prestige Smart System; Walgreens). The rats were then injected subcutaneously at the back of the neck with 0.5 units/kg of Actrapid® insulin (Novo Nordisk; U.S.A). Subsequently, blood glucose levels were measured serially at times 15, 30, 60 and 120 minutes after the insulin injection.

**Bodyweight and adiposity:** the rats were weighed daily before drug administration. At the end of 4 weeks, the rats were euthanised using an intraperitoneal injection of Ketamine (80mg/kg) and Xylazine (10mg/kg). Interscapular, inguinal, and epididymal fat depots were then carefully excised and weighed to measure adiposity.

**Statistical analysis:** experimental data were statistically analysed using STATA 16.0 statistical package with the significance level set at p<0.05. The Shapiro Wilk test determined normalcy. Normally distributed data were analysed by one way ANOVA while the Kruskal Wallis test was used to analyse non-normally distributed data. In cases of significance, posthoc analysis was performed by either the Tukey's honestly significant difference (HSD) test or the Dunn post hoc test for normally and non-normally distributed data respectively.

## Results

**Insulin sensitivity:** we determined insulin sensitivity by calculating the area under the curve (AUC) of the graphs obtained by plotting blood glucose concentration against time following a two-hour insulin tolerance test. Subsequently, AUC was calculated using the composite trapezoidal method by applying the pksumm and the pkexamine commands in STATA. Analysis of the AUCs found a statistically significant difference in insulin sensitivity between the treatment groups α^2^ (2) = 6.080, p = 0.0478, with a mean rank AUC of 60.00 for the LPV/r+AZT group, 32.00 for LPV/r+AZT+rimonabant and 28.00 for controls. A Dunn's pairwise comparison test followed, revealing that, insulin sensitivity was significantly higher in rats treated with LPV/r +AZT + rimonabant compared to rats treated with LPV/r+AZT only (329.14 ± 85.19 vs 389.81 ± 37.96, p = 0.0238) (Table 1). There was no statistical difference in insulin sensitivity between rats treated with LPV/r+AZT+rimonabant and control rats (p=0.39).

**Table 1:** values for area under curve for blood glucose concentration against time graphs for each rat in the different experimental groups

Rat	Area under curve		
	LPV/r+AZT*	LPV/r+AZT+Rimonabant*	Controls
1	345.108	307.696	335.941
2	381.112	478.207	298.821
3	450.332	303.791	342.171
4	387.461	293.456	302.378
5	385.038	307.696	279.156

Insulin sensitivity was determined by plotting blood glucose concentration against time following a two-hour insulin tolerance test and then calculating the area under the curve for each graph. Dunn's pairwise comparison test revealed that rats treated with LPV/r+AZT+Rimonabant were more insulin sensitive than rats treated with LPV/r+AZT only, *p = 0.0238. LPV/r: Lopinavir/ritonavir, AZT: Azidothymidine, AUC: Area Under Curve

**Figure 1:**
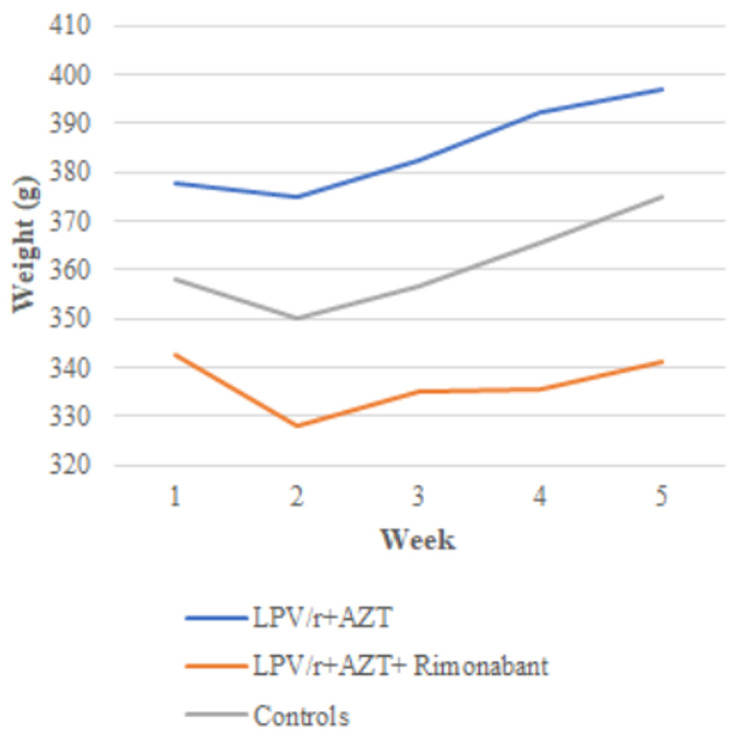
weekly weight gain graph

**Bodyweight and adiposity:** there were no differences in body weight between the treatment groups at the beginning of the study (p=0.129). All the rats gained weight during the study (Figure 1), however, after four weeks of treatment, there was a statistically significant difference in body weight between groups determined by one-way ANOVA (F(2,12) = 4.07, p = 0.045). A Tukey post hoc test revealed that rats treated with LPV/r + AZT + rimonabant weighed significantly less (339.6 ± 22.65 g vs 398.8 ± 50.25 g) than rats treated with LPV/r + AZT only (Table 2). There was no statistically significant difference in weight between LPV/r + AZT + rimonabant treated rats and control rats (339.6 ± 22.65 vs 371.6 ± 13.96 g).

**Table 2:** mean body weights (g) at the start and the end of the study

Treatment group	N	Mean Start Weight (g)	Mean End Weight (g)
LPV/r+AZT	5	378.6 ± 59.62	398.8 ± 50.25*
LPV/r+AZT+Rimonabant	5	324.2 ± 23.08	339.6 ± 22.65*
Controls	5	353.0 ± 21.86	371.6 ± 13.96

Values represented as mean ± SD.* *p = 0.045, LPV/r: Lopinavir/ritonavir, AZT: Azidothymidine, SD: Standard Deviation

We determined adiposity by carefully excising and weighing interscapular, inguinal, and epididymal fat depots (Table 3). Rats treated with LPV/r+AZT+rimonabant had 58% less adiposity than rats treated with LPV/r+AZT-only (5.58 ± 1.83 vs 9.59 ± 3.72 g). They also had 32% less adiposity than control rats, (5.58 ± 1.83 vs 8.21 ± 1.32 g). These differences were not statistically significant by one-way ANOVA (p=0.073).

**Table 3:** weights of fat depots

Groups	Mean fat depot weight (g)
LPV/r+AZT	9.59 ± 3.72*
LPV/r+AZT+Rimonabant	5.58 ± 1.83*
Controls	8.21 ± 1.32*

Values represented as mean ± SD.*, p = 0.073, LPV/r: Lopinavir/ritonavir, AZT: Azidothymidine, SD: Standard Deviation

## Discussion

In this experimental study, co-administration of the cannabinoid type 1 receptor blocker, rimonabant, with antiretroviral drugs known to produce insulin resistance (LPV/r+AZT), resulted in significantly higher insulin sensitivity in Sprague Dawley rats as determined by calculation of area under curve of graphs of blood glucose concentration plotted against time following a two-hour insulin tolerance test. The rats also had lower body weight. Besides, CBR1 antagonism resulted in smaller fat depots and leaner body composition.

The study design employed was simple and easy to replicate, while the in-vivo experiments mimic routine drug administration in the real world. The main limitation of the study is the use of the insulin tolerance test (ITT) in determining insulin sensitivity rather than the gold standard hyperinsulinemic-euglycemic clamp (HEC). Besides, the clinical use of first-generation CB1R antagonists such as rimonabant has been suspended due to adverse central effects [18]. However, promising second-generation CBR1 antagonists with little blood-brain barrier penetration are in development [19].

In previous studies, CB1R antagonism has proven benefits on insulin sensitivity [20], however, to our knowledge, this is the first study to look into the effects of CB1R antagonism in the context of antiretroviral drug administration. Similar to the evolution of type II diabetes mellitus in the general population, both PIs and NRTIs induce insulin resistance by actions at multiple tissue targets in a multifactorial and polygenic manner [11]. In adipocytes, PIs inhibit GLUT 4 in a non-competitive dose-dependent manner while in pancreatic islets, they inhibit insulin secretion [21]. NRTI treatment, on the other hand, reduces whole-body glucose disposal [12]. Overactivity of the endogenous cannabinoid system (ECS) occurs in hyperglycaemic and obese states [5]. The mechanisms by which PIs and NRTIs induce insulin resistance appear to overlap significantly with those mechanisms activated by an overactive ECS [22]. In the present study, we hypothesised that endogenous cannabinoid activity is involved in PI and NRTI induced insulin resistance, and we demonstrated that blockade of the ECS ameliorates the insulin resistance produced by chronic treatment with the PIs Lopinavir/ritonavir alongside the NRTI, Zidovudine. The implication of these findings is that cART drugs may work cooperatively with or via the ECS to produce deleterious metabolic and physical changes.

Future studies can measure by direct assay, changes in endogenous cannabinoid activity directly either as a result of treatment with antiretroviral drugs in similar study designs or in HIV patients who present metabolic derangements while on treatment with cART. Besides, cannabinoid agonism via Dronabinol, or Δ-9-tetrahydrocannabinol, a synthetic mimic of *Cannabis Sativa L*., has been used to treat HIV induced anorexia successfully [23]. This observation presents an opportunity to study the nexus between caloric intake and endogenous cannabinoid tone in HIV infected patients with or without metabolic derangements.

## Conclusion

In conclusion the present study indicates that endogenous cannabinoid tone is probably involved in the etiology of insulin resistance and disordered adiposity that accompanies use of Lopinavir/Ritonavir and AZT containing cART regimens. Enhanced endogenous cannabinoid tone is a pathophysiologic mechanism shared with metabolic syndromes of other etiologies. The study, therefore, also points to a potentially viable new approach for investigating root causes and managing insulin resistance associated with cART which still remains unsatisfactory and is a leading cause of morbidity and mortality in HIV patients.

### What is known about this topic

- *Overactivity of the endogenous cannabinoid system is a crucial orchestrator of metabolic disease and obesity;*
- *CBR 1 receptor antagonism ameliorates many of these metabolic derangements and physical changes resulting in better insulin sensitivity, lower body weight and leaner body composition in both human and animal studies*.

### What this study adds

- *Insulin sensitivity is improved by treatment with the CBR 1 receptor antagonist, rimonabant in the context of treatment with antiretroviral drugs that are known to induce insulin resistance such as Lopinavir/ritonavir and zidovudine;*
- *Administering the CBR 1 receptor antagonist rimonabant, along with antiretroviral drugs known to induce lipodystrophy results in a leaner body composition; these findings suggest a role for the endogenous cannabinoid system in the evolution of antiretroviral therapy induced metabolic syndrome;*
- *Future study designs can measure directly, endogenous cannabinoid activity in response to administration of cART in animal models and in HIV patients on cART who present with metabolic derangements and lipodystrophy*.
